# A High-Performance Optoelectronic Sensor Device for Nitrate Nitrogen in Recirculating Aquaculture Systems

**DOI:** 10.3390/s18103382

**Published:** 2018-10-10

**Authors:** Cong Wang, Zhen Li, Zhongli Pan, Daoliang Li

**Affiliations:** 1 College of Information and Electrical Engineering, China Agricultural University, Beijing 100083, China; ouyangyucong2005@163.com (C.W.); leezn2009@163.com (Z.L.); 2 Key Laboratory of Agricultural Information Acquisition Technology, Ministry of Agriculture, Beijing 100083, China; 3 Beijing Engineering and Technology Research Centre for Internet of Things in Agriculture, Beijing 100083, China; 4 Healthy Processed Foods Research Unit, USDA-ARS-WRRC, 800 Buchanan St., Albany, CA 94710, USA; zlpan@ucdavis.edu; 5 Department of Biological and Agricultural Engineering, University of California, One Shields Avenue, Davis, CA 95616, USA

**Keywords:** nitrate nitrogen, buffer operator, absorbance

## Abstract

The determination of nitrate nitrogen (NO_3_-N) in recirculating aquaculture systems is of great significance for the health assessment of the living environment of aquatic animals. Unfortunately, the commonly used spectrophotometric methods often yield unstable results, especially when the ambient temperature varies greatly in the field measurement. Here, we have developed a novel handheld absorbance measurement sensor based on the thymol-NO_3_-N chromogenic rearrangement reaction. In terms of hardware, the sensor adopts a dual channel/dual wavelength colorimeter structure that features a modulated light source transmitter and a synchronous detector receiver. The circuit measures the ratio of light absorbed by the sample and reference containers at two LEDs with peak wavelengths at 420 nm and 450 nm. Using the modulated source and synchronous detector rather than a constant (DC) source eliminates measurement errors due to ambient light and low frequency noise and provides higher accuracy. In terms of software, we design a new quantitative analysis algorithm for absorbance by studying colloid absorbing behavior. The application of a buffer operator embedded in the algorithm makes the sensor get the environmental correction function. The results have shown that the sensitivity, repeatability, precision and environmental stability are higher than that by ordinary spectrophotometry. Lastly, we have a brief overview of future work.

## 1. Introduction

With the increase in land and water costs, the government’s strict control of the discharge of aquaculture wastewater and the market’s strong demand for healthy aquaculture products, aquaculture is gradually developing towards a more intensive and environmentally friendly direction. Recirculating aquaculture systems (RAS) are receiving more and more attention and have become a complementary and alternative industry to traditional pond aquaculture in developed countries. RAS are growing in popularity because of some key features, including enhanced biosecurity, high levels of control, the ability to grow marine animals away from the coast, and the ability to grow warm-water species indoors practically anywhere [1,2]. In RAS, animals break down proteins in the feed, about 20–25% accumulates in the animal tissues and the remainder is excreted into the water as ammonium and organic nitrogen [3]. Through conventional water treatment technology (precipitation, filtration and biological purification), the organic matter in the aquaculture water can be effectively removed [4], and the ammonia nitrogen is converted to nitrate nitrogen (nitrification), so that the water quality can be improved to a certain extent. However, nitrate accumulation occurs in the absence of denitrification when RAS are operated with minimal water exchange or dilution [5].

Nitrate is much less toxic than either ammonia or nitrite; however, high concentrations and long exposure times reduce animal growth and can decrease survival. Freshwater animals appear to be more sensitive to nitrate than marine animals [6,7,8,9]. David et al. (2010) found that growth rates and feed intake decreased for Pacific white shrimp exposed to nitrate nitrogen (NO_3_-N) concentrations >220 mg/L [10]. Hamlin reported that in RAS, as Siberia sturgeon grew larger, they became more sensitive to NO_3_-N concentration [11]. Approximately 125 mg/L of NO_3_-N would affect the growth and health status of juvenile turbot (*Psetta maxima*) [12]. Several studies [13,14] have indicated that NO_3_-N can also significantly delay the spawning time of *Oryzias latipes* and reduce the spawning number at concentrations of 50 mg/L. Even 1 mg/L NO_3_-N is fatal to *Penaeus monodon* protozoa. In a study evaluating the potential effect of 200 mg/L NO_3_-N on hybrid striped bass *Morone chrysops × M. saxatalis*, Hrubec (1996) reported increased mortality, decreased immune function, and physiological changes consistent with pathology such as gill hyperplasia and blood chemistry alterations [15]. Recently, Davidson et al. (2011) suggested that approximately 100 mg/L NO_3_-N was a potential causative agent of abnormal rainbow trout swimming behaviors such as rapid swimming velocity and side swimming, and that NO_3_-N concentrations >400 mg/L were potentially related to more severe physiological effects such as spinal deformities and increased mortality [16]. In addition, Schram et al. (2014) found that feed intake and growth rates decreased for African catfish *Clarias gariepinus* exposed to NO_3_-N concentrations >140 mg/L [17]. Several other studies have indicated that NO_3_-N can be chronically toxic to salmonid eggs and fry at concentrations <200 mg/L with sub-lethal effects occurring at <25 mg/L [18,19]. Therefore, tank-side/field measurements may be important to ensure that RAS are optimized to maintain NO_3_-N below an upper threshold. Several strategies exist for dealing with nitrate, including water exchange, phytoremediation, denitrification, and heterotrophic assimilation. System managers should consider these carefully, and in many cases, the solution to nitrate accumulation may prove to be a combination of strategies put together in a cost-effective manner.

Numerous methods have been reported for the detection and determination of nitrate, including Cu/Cd column reduction [20,21,22], the phenol-two-sulfonic acid method [23,24], gas-phase molecular absorption spectrometry [25,26,27], ion chromatography [28,29] and ultraviolet spectrophotometry [30,31,32,33]. The traditional Cu/Cd column methods continue to hold a dominant position due to its low cost and easy feasibility. However, this method is often time-consuming and suffers from low results and interference from other ions. The use of Cu/Cd powder is dangerous as it is easy to cause heavy metal pollution. The stability and reproducibility of the phenol-two-sulfonic acid method is poor. In addition, chloride and nitrite ions will seriously interfere with the determination. Ion chromatography and gas-phase molecular absorption spectrometry have the advantage of high sensitivity, but the requirements for sample preparation and derivatization procedures and specialized equipment make these methods more expensive than spectroscopic and electrochemical methods. When natural waters containing a large amount of organic matter, nitrite, hexavalent chromium and bromide are determined by the ultraviolet spectrophotometry method, the error is obvious and the blank value of the water body is high, which will cause serious interference. If two times the absorbance of the water sample at 275 nm wavelength is larger than 10% of the absorbance at 220 nm wavelength, this method will no longer be applicable.

In this paper, we develop a high-performance optoelectronic sensor device based on the chromogenic principle of nitrate derivatives. The transducer is composed of a group of optical elements, a set of signal conditioning circuit boards and a set of system control panels. Our proposed model optimizes the analysis of samples with a higher sensitivity, lower method detection limit and environmental correction function. Moreover, the addition of masking agents eliminates the influence of potential chloride and nitrite nitrogen on the measurement.

We begin by discussing the detection principle and the modeling method, then describe the details of the sensor design considerations and the design of hardware circuit, and then report the reagents and analytical procedures. Finally, the optoelectronic sensor device is evaluated from the aspect of sensitivity, repeatability, precision and environmental reliability.

## 2. Sensor Principles and Modeling Method

At certain concentrations of sulfuric acid medium, thymol (2-isopropyl-5-methylphenol) reacts with nitrate to form nitro-phenol compounds. When the test solution is adjusted to alkaline, molecular rearrangement will take place to form yellow compounds. The color intensity of the compound is directly proportional to the amount of nitrate. The main reactions are as follows:

$$\begin{array}{l} (CH_{3})(C_{3}H_{7})C_{6}H_{3}OH+H_{2}SO_{4}+NO_{3}^{-}\to (CH_{3})(C_{3}H_{7})(NO_{2})C_{6}H_{2}OH+HSO_{4}^{-}+H_{2}O\\ (CH_{3})(C_{3}H_{7})(NO_{2})C_{6}H_{2}OH+NH_{3}\cdot H_{2}O\to (CH_{3})(C_{3}H_{7})(N_{2}O_{2}H_{4})+H_{2}O \end{array}$$

**Figure 1:**
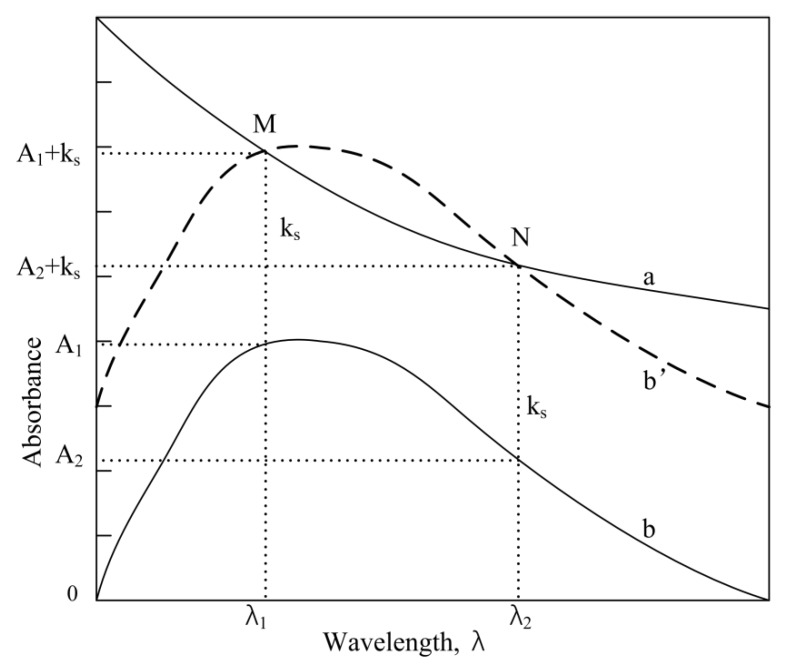
Absorption spectra sketch: a, suspension particle liquid against water; b, absorption spectrum of a color solution; b’, the same as curve b but the absorbance was shifted up by k_s_ distance; *λ*_1_, peak absorption (M point) at primary wavelength; *λ*_2_, valley absorption (N point) at secondary wavelength.

For a suspension liquid or a colloid solution, the absorbance (*A*) is relative to the incident wavelength (*λ*) and the particle’s property parameters (*ϕ*, *ε*). In Figure 1, curve a shows the absorption spectrum sketch. Their mathematical expression is followed by [34]:

(1) $$A=\phi \varepsilon^{-2}\lambda^{-\varepsilon }\;$$

Curve b in Figure 1 gives the absorption of a color solution. We can see that if curve b moves up k_s_ distance to the b’ position, the cross-points (both M and N) are formed in curve a (b//b’). At the wavelengths of *λ*_1_ and *λ*_2_, the amount of the absorption substance of the chromogenic fluid to the light equals the amount of the absorption substance of suspended particles to the light. Therefore,

(2) $$\left\{ \begin{array}{l} A_{1}+k_{s}=k_{2}\lambda_{1}^{-m}\\ A_{2}+k_{s}=k_{2}\lambda_{2}^{-m} \end{array}\right.\;$$

Both *k*_1_ and *k*_2_ are the calculation factors. A lot of experiments have shown that the factor M is an exponent function with ligand amounts, that is with the determined component concentration (*x* mg/L) when *k_s_* = 1.

(3) $$m=\alpha x^{\beta }\mathrm{or}\log m=\beta \log x+\log\alpha \;$$

Both *α* and *β* are the regression coefficients calculated from a standard series of the determined component. Once the wavelengths *λ*_1_ and *λ*_2_ are selected, their values are constant.

In order to make the analysis have the highest sensitivity, the wavelength λ_1_ should be selected at the maximum absorption wavelength, and it is defined as the primary wavelength (*λ_p_*):*λ_p_* = *λ*_max_. Wavelength *λ*_2_ can be selected arbitrarily, but different *λ*_2_ corresponds to variable α and β values, and in general *λ*_2_ > *λ*_1_, which makes M > 0. Taking into account the contrast of the absorbance between the two wavelengths, it is advisable to choose the wavelength position at half the maximum absorption (0.5 *A*_max_), termed as secondary wavelength (*λ_s_*). The following relationship is further obtained:

(4) $$\frac{A_{p}+1}{A_{s}+1}={\left(\frac{\lambda_{p}}{\lambda_{s}}\right)}^{-m}\;$$

where *A_p_* and *A_s_* are the primary and secondary absorbance values corresponding to wavelengths *λ_p_* and *λ_s_*, respectively. From Equation (4), we may obtain the M value to calculate the X value from Equation (3). Both *A_p_* and *A_s_* will change synchronously when the operating environment changes (for example, ambient temperature), but the calculated value of the buffer function (*A_p_* + 1)/(*A_s_* + 1) changes little. Therefore, Formula (4) has the correct function for operation conditions so that both α and β remain almost constant in the M(X) model.

## 3. Experimental Details

### 3.1. Sensor Design Considerations

**Figure 2:**
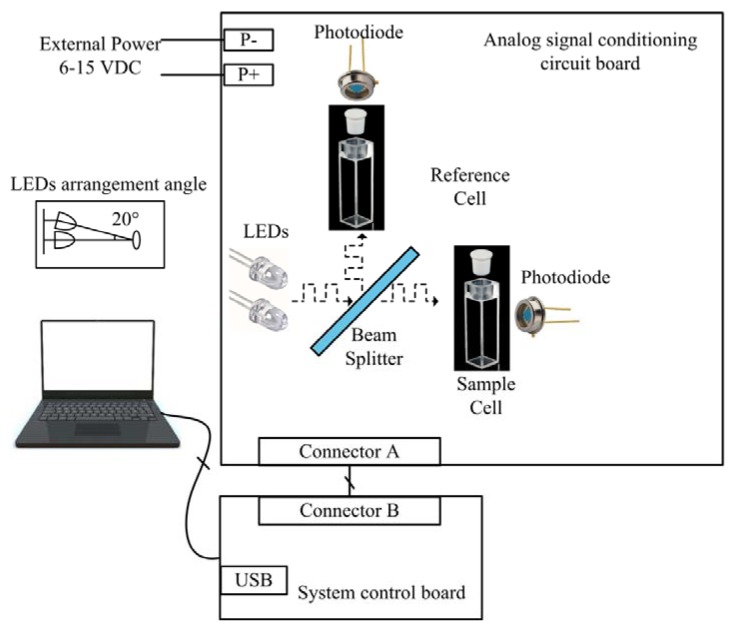
Function block diagram of test system.

The overall structure design of the detection system is shown in Figure 2. The transducer is composed of a group of optical elements, a set of signal conditioning circuit boards and a set of system control panels. The signal conditioning circuit board includes two identical signal processing channels (reference channel and sample channel). Both of the channels receive the transmitted light signal, and successively conduct photoelectric conversion, amplifying, filtering, reshaping, and analog-to-digital conversion. The system control board is responsible for driving the analog-to-digital converter chip, triggering the light source and the synchronous rectifier, and adjusting the programmable gain. In addition, it also models the collected digital signals and uploads the calculation results to the host computer through the USB interface.

The excited light source at a specific wavelength is irradiated to the surface of the beam splitter in the form of modulation, and the beam splitter delivers half of the incident light into the reference container and the other half into the sample container. Transmission lights fall into the photoelectric detection devices through reference and sample containers. The amplitude of the photoinduced current depends on the absorbance of the media characteristics in the containers. Three operations including current-to-voltage conversion, signal shaping and analog-to-digital conversion are carried out to guarantee the accuracy of data modeling by the microprocessor.

Using the second reference channel to calibrate the luminous flux of the light source in real time can effectively eliminate systematic errors caused by environmental temperature fluctuations and provides higher detection accuracy. In other areas of sensor research and development, for example, using MEMS and optical type and plasmonic sensors, similar methods such as differential measurement to improve sensor performance have been reported [35]. In addition, similar signal processing (implemented in postprocessing modes or in electronic circuits) can often improve the signal-to-noise ratio dramatically [36,37].

#### 3.1.1. Incident Light Drive Circuit

**Figure 3:**
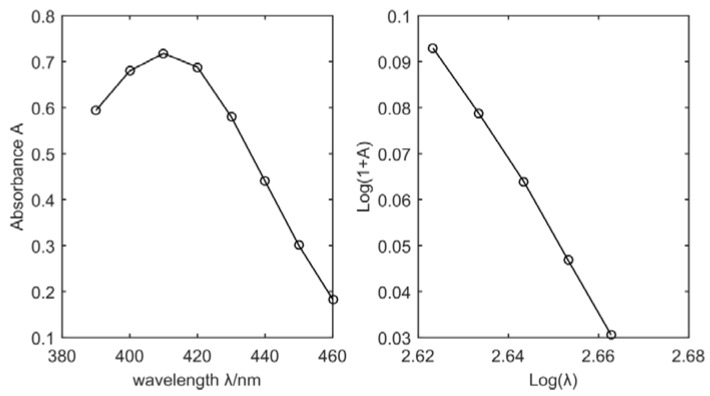
Absorption spectra of the color solution and the Log(1 + A) − Log(λ) curve.

A certain concentration of NO_3_-N standard solution was selected for the experiment, and the absorption spectrum of the color solution is shown in Figure 3. In the wavelength range of 420–460 nm, the curve of Log(1 + A) − Log(λ) presents a good linear relationship (the result of d(*log(1 + A))*/d(*log(λ))* is a constant). Therefore, when the concentration of NO_3_-N is a constant value, even if any two wavelengths from 420 nm to 460 nm are chosen, the M value calculated by Equation (4) is also a constant.

To improve the flexibility of the system’s optical and mechanical design, and considering the requirement for the wavelength of absorption light to color solution, the system uses a L420R-01 (420 nm), L450R-01 (450 nm) InGaN super bright LED lamp produced by Epitex (Japan) as the main source and secondary light source of the instrument. According to the product handbook, the maximum forward current can reach 100 mA, the spectral half width is 19 nm, the radiant intensity is 220 mW/Sr, the viewing half angle is ±8 degrees, and the performance basically meets the requirements of portable instruments.

**Figure 4:**
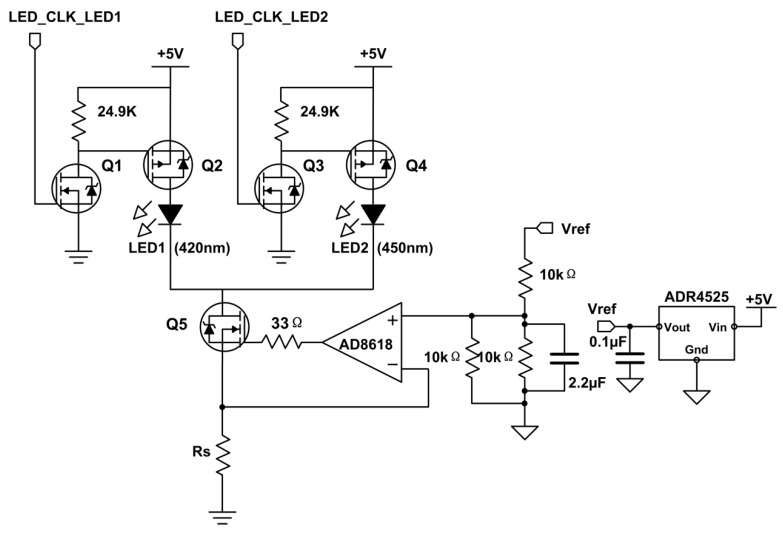
LED constant current source drive control circuit. LED_CLK_LED1, LED1 control signal; LED_CLK_LED2, LED2 control signal; Vref, standard reference voltage.

The two LEDs are vertically stacked and irradiated uniformly on the beam splitter mirror surface. In order to reduce the temperature drift and prolong the service life of the LEDs, an integrated operational amplifier AD8618 and a Field Effect Transistor Q5 (N-channel enhancement mode, 2N7002) were selected to form a constant current control circuit with closed loop negative feedback control to keep the constant flow of the LED’s current (See Figure 4). The driver current directly depends on the ratio between the output of the reference power chip (ADR4525, low noise voltage references featuring ±0.02% maximum initial error) and the sampling resistance, Rs. To improve the radiant intensity of the light source and save the energy consumption of the instrument, the pulse modulation mode is used to drive the light source. This operation mode can ensure that the light intensity in a single period can be increased without increasing the average current; thus, the absorption effect of the liquid phase is enhanced and the sensitivity of the device is improved. When the Gate of Q1 (2N7002) is input high, Q1 is turned on. At this moment, the Gate of Q2 (P-Channel Enhancement MOSFET, Si2301DS) is at low level, Q2 is turned on and LED1 is opened. The LED1 can be closed by pulling down the input of the Q1.

#### 3.1.2. Signal Conditioning Circuit

The distribution of nitrate in natural waters shows that there is a very low value on the absorption of the incident light in the color solution, so the sensitivity of the photoelectric sensor is very high. The accuracy of the system is determined by the minimum limit of the photoelectric sensor that can capture the transmission light signal. Therefore, the photoelectric conversion module is an important part of the system.

This system uses two S1336-44BK photodiodes (Hamamatsu) as optical sensors. This photodiode has high sensitivity, good linear characteristics and a fast reaction speed. When receiving the light signal, it will produce a current that changes with the intensity of the light signal.

**Figure 5:**
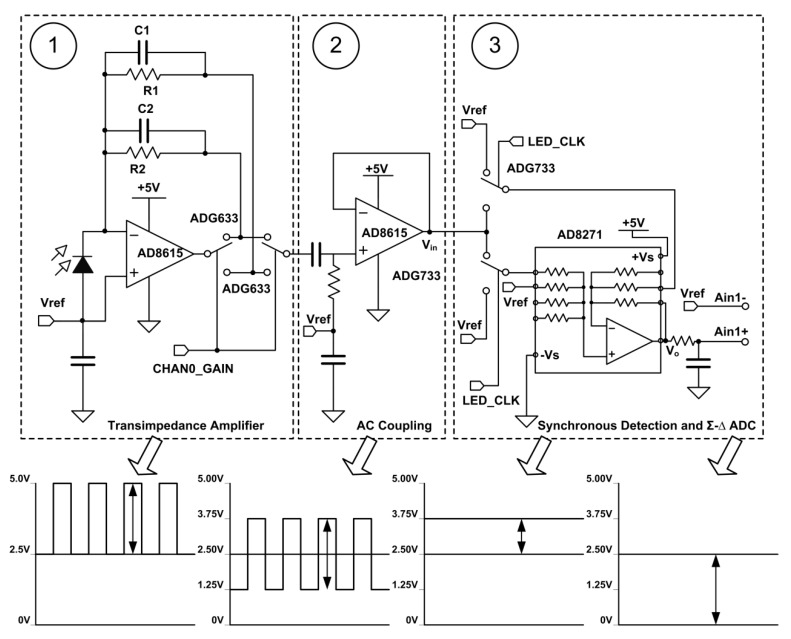
Signal conditioning circuit diagram and time-domain waveforms at each step. CHAN0_GAIN, channel 0 gain control signal; LED_CLK, LED pulse control signal; V_in_, ADG733 input signal; V_o_, AD8271 output signal; Ain1-, Ain1+, differential input mode.

Figure 5 contains a typical I/V conversion circuit that connects two conversion resistors and two input compensation capacitances between an operational amplifier output and the inverse input terminal, while the forward input is biased at the 2.5 V level. The output of the first stage operational amplifier equals the product of the conversion current of the photodiode and the conversion resistance plus the bias voltage. To adapt the absorbance response to different concentrations of the chromogenic liquid, the system dynamically adjusts the gain of the transfer resistance (R1 or R2) through a set of single-pole double-throw switches (ADG633). High precision AD8615 is chosen as the primary operational amplifier because of its low offset voltage (65 uV), low noise (8 nV/√Hz) and low input bias current (1 pA).

The converted voltage signal contains the unwanted output offset voltage and low frequency ambient light noise, which needs to be filtered through a simple buffer AC coupler and the filter cut-off frequency is set at 10 Hz. The input of the second stage operational amplifier is still biased at the 2.5 V level.

The next stage of the circuit is a synchronous rectifier, which only allows the signals synchronized with the LED clock to pass through. It looks like a narrow band filter and importantly plays the role of AC–DC converter under the control of a group of analog switches (ADG733). The function configuration of ADG733 is listed in Table 1. The processing result of the synchronous rectifier is finally transmitted to the differential input end of the analog-to-digital conversion chip (AD7798, programmable gain amplification factor (PGA) = 2).

#### 3.1.3. The Front-End Circuitry Noise Analysis

**Figure 6:**
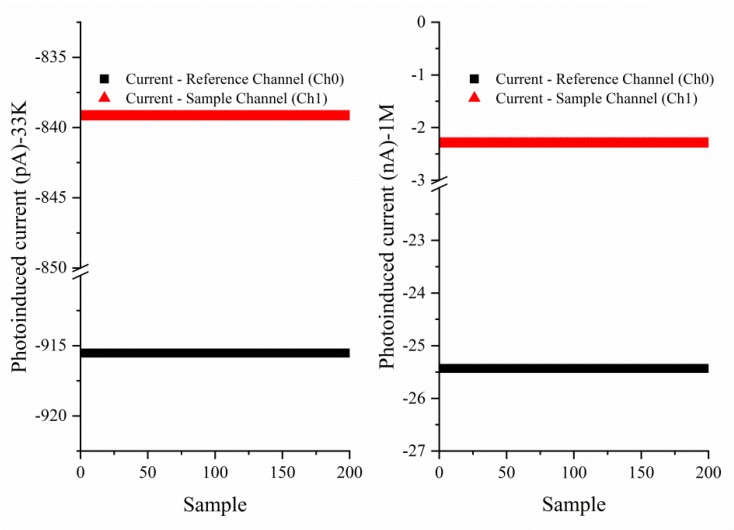
Front-end circuitry noise under two kinds of gain settings.

We acquired data with all LEDs disabled, while keeping the synchronous rectifier still working at the clock frequency of the LEDs. However, the transducer will not detect the transmission light at the same frequency. In this way, we can analyze the noise pollution level of the integrated operational amplifier and the analog-to-digital converter to the front-end circuit. Figure 6 shows that the measured noise voltages range from −76 μV to −915 μV, which falls exactly into the AD8271’s expected distribution of input offset voltage error. Experiments indicate that the front-end circuit does not contribute significant noise to the whole system.

**Figure 7:**
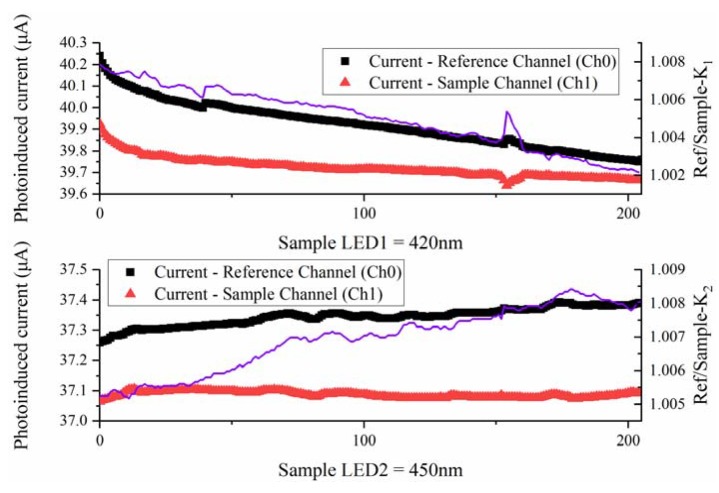
The correction coefficient readings at two wavelengths.

When traditional colorimeters were used to measure a large number of samples, only one blank test was usually conducted at the beginning of the measurement to estimate the luminous flux of the excitation source. The calculation process of the absorbance of all samples was carried out against this blank sample. If the ambient temperature fluctuated, the luminous flux of the light source would also change, and the system measurements deviated. To address this problem, we had installed the reference light path to calibrate the luminous flux of the incident light in real time. We added a certain amount of distilled water to the two containers and made the ratio between the reference-photocurrent (I_R_) and sample-photocurrent (I_s_) as the correction coefficient (K). Figure 7 shows the correction coefficient readings from 205 samples. We defined constants *K*_1_ (*K*_1_ = 1.005) and *K*_2_ (*K*_2_ = 1.007) as the correction factors of LED1 and LED2, respectively. The corrected absorbance is further obtained:

(5) $$A_{p}=\log\frac{V_{R420}}{K_{1}V_{S420}}\;$$

(6) $$A_{s}=\log\frac{V_{R450}}{K_{2}V_{S450}}\;$$

where *V_R_*_420_ and *V_R_*_450_ are the reference channel output voltages at wavelength 420 nm and 450 nm and *V_S_*_420_ and *V_S_*_450_ are the sample channel output voltages at the above wavelengths, respectively.

### 3.2. Reagents and Analytical Procedures

#### 3.2.1. Reagents

All the chemical reagents used in this experiment were purchased from Shanghai Macklin Biochemical Co., Ltd. (Shanghai, China). The purity of the reagents was at least analytical grade. The freshly deionized water was used as a general solvent.

##### Ammonium Sulfamate Solution

Ammonium sulfamate solution (20 g/L) was made by dissolving 2.0 g ammonium sulfamate (NH_4_SO_3_NH_2_) in 100 mL acetic acid solution (1 + 4). We shook well the mixed solution to make it fully dissolve.

##### Thymol–Ethanol Solution

Thymol-ethanol solution (5 g/L) was prepared by adding 0.5 g thymol ((CH_3_)(C_3_H_7_)C_6_H_3_OH) to 100 mL absolute ethanol. We shook well the mixed solution to make it fully dissolve.

##### Silver Sulfate–Sulfuric Acid Solution

Silver sulfate-sulfuric acid solution (10 g/L) was made by dissolving 1.0 g silver sulfate (Ag_2_SO_4_) in 100 mL sulfuric acid (standard density, 1.84 g/mL). The prepared solution was stored in the dark at room temperature.

##### Nitrate Nitrogen Standard Solution

NO_3_-N standard solution (ρ(NO_3_-N) = 1 g/L) was prepared by dissolving 6.071 g sodium nitrate (NaNO_3_) in 1000 mL freshly deionized water. Sodium nitrate needed to be dried at 105–110 °C for an hour in advance. We added 2 mL chloroform to it for preservation. NO_3_-N working solution (ρ(NO_3_-N) = 10 mg/L) was made daily by pipetting 5.00 mL standard solution into 500 mL deionized water.

#### 3.2.2. Basic Procedures

1.0 mL of filtered water samples were collected in dry 50 mL colorimetric tubes. Ammonium sulfamate solution (0.1 mL) was added into the colorimetric tube, shaken well and incubated for 5 min. We carefully dropped 0.2 mL of thymol-ethanol solution into above solutions along the center of the colorimetric tube, then kept shake and pipetted 2 mL of silver sulfate-sulfuric acid solution to the colorimetric tube, incubated for 5 min. Finally, we added 8 mL of deionized water, mixed evenly and then dropped 9 mL of ammonium hydroxide solution (standard density, 0.88 g/mL) to trigger the compounds molecular rearrangement. Now the precipitate of silver chloride is dissolved. We added deionized water to 25 mL scale of colorimetric tube and mixed evenly.

In the experiment, the interference of the chloride ions was eliminated by silver sulfate, and silver chloride precipitate was formed and dissolved into ammonia water to form a complex. Ammonium sulfamate eliminated the interference of coexisting nitrite nitrogen.

We took two 1 cm capped plastic cuvettes, one as a reference container to fill the blank solution (freshly deionized water), and the other as a sample container to fill the sample or standard solutions. Through the absorbance sequence values at the wavelength 420 nm and 450 nm, we built regression equation and got the content of nitrate nitrogen in the samples.

Table 2 lists the parameter description of traditional model and proposed model. A typical function model was a linear function model, written in matrix.

(7) $$y=b_{0}t+b_{1}$$

(8) $$\underset{b0,b1}{\min}{\left\|\left(\begin{array}{cc} 1 & t_{1}\\ \vdots & \vdots \\ 1 & t_{n} \end{array}\right)\left(\begin{array}{c} b_{0}\\ b_{1} \end{array}\right)-\left(\begin{array}{c} y_{1}\\ \vdots \\ y_{n} \end{array}\right)\right\|}_{2}=\underset{b}{\min}{\left\|Ab-Y\right\|}_{2}$$

(9) $$b_{1}=\frac{\sum_{i=1}^{n}\left(t_{i}-\bar{t})(y_{i}-\bar{y}\right)}{\sum_{i=1}^{n}{\left(t_{i}-\bar{t}\right)}^{2}},b_{0}=\bar{y}-b_{1}\bar{t},\bar{t}=\frac{1}{n}\sum_{i=1}^{n}t_{i}$$

*K*_1_, calibration coefficient for LED1 luminous flux; *V_S_*_420_(*n*), sample channel output voltage when the *n*-th standard solution is measured; *V_R_*_420_(*n*), reference channel output voltage when the *n*-th standard solution is measured; *M_n_*, the calculation procedure is shown in Equation (4); *x_n_*, the concentration of the *n*-th standard solution.

### 3.3. Transducer Performance Test

**Figure 8:**
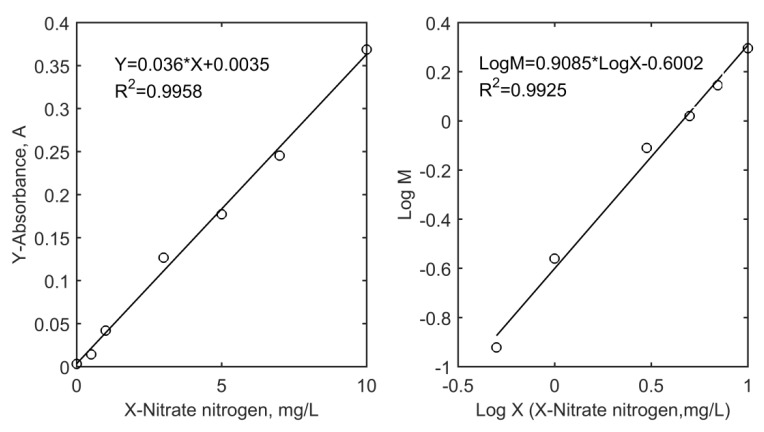
Traditional model curve and new model curve.

We took seven colorimetric tubes with addition of 0, 0.05, 0.10, 0.30, 0.50, 0.70 and 1.00 mL of NO_3_-N working solutions and diluted them to 1 mL with fresh deionized water. We sequentially added reaction reagents and measured the absorbance of the final color solutions. The plastic cuvettes filled with color solutions need to be gently shaken to remove bubbles from the solution and the outside surface of the cuvettes also need to be kept clean. Figure 8 shows the calibration curves obtained by the traditional model and the proposed model. The calculation of variable M is based on Equation (4). The regression result LogM = 0.9085 × LogX − 0.6002 can be transformed to the calculation model of nitrate nitrogen, which is X = 4.577 × M^1.101^. The slope of the proposed method is 0.9085, being 25 times that of the traditional method. The proposed method adopts function (A_p_ + 1/A_s_ + 1) to quantify nitrate nitrogen, which changes the quantized form of single variable (A_p_), and the buffer performance improves the sensitivity of the analysis.

**Figure 9:**
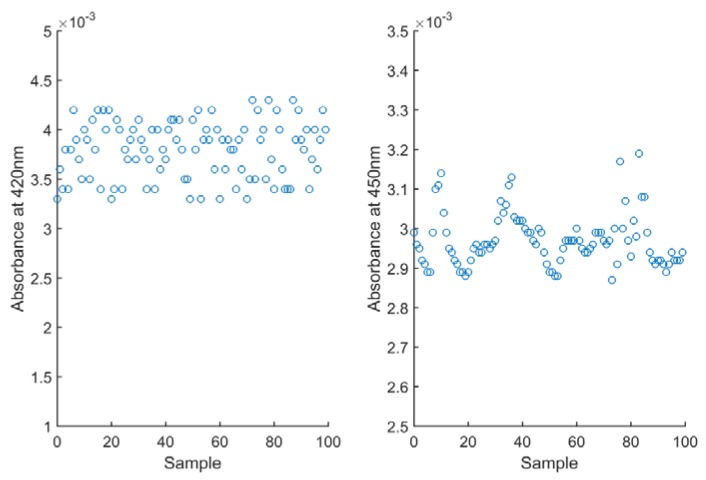
The absorbance of the blank working solution at 420 nm and 450 nm.

To calculate the detection limit (LOD) of the two detection methods, we filled the sample container with 0 mg/L NO_3_-N working solution. One hundred data points were recorded continuously as shown in Figure 9 and used to calculate the standard deviation value. The method detection limit, estimated as three times the standard deviations of the blank, was 0.036 mg/L. The calculation procedures are as follows: LOD = 4.577 × (3 × S_d_/K)^1.101^, where S_d_ is the standard deviation of the blank signal M value and K is the transducer calibration curve slope (S_d_ = 0.012299, K = 0.9085). The result is a little lower than that of the traditional method (0.085 mg/L).

**Figure 10:**
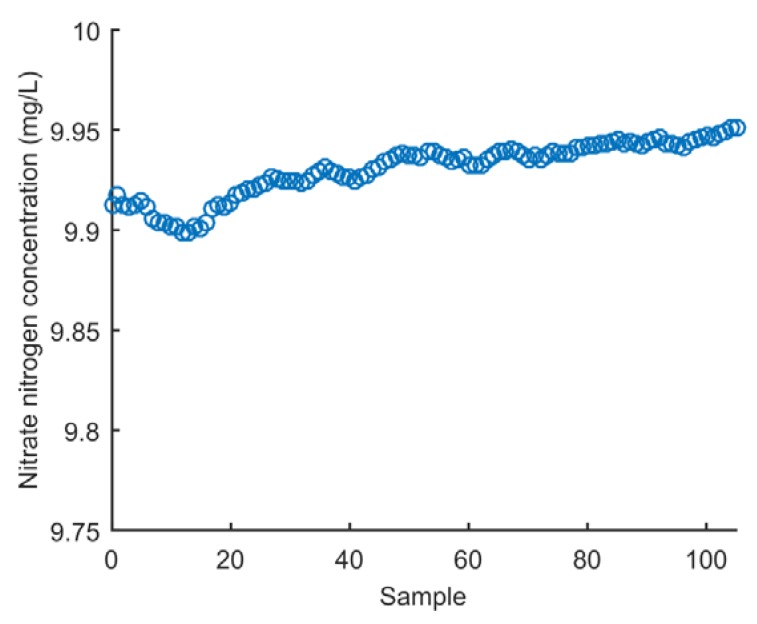
Repeatability of the measurements.

The repeatability of measurements refers to the variation in repeat measurements made on the same subject under identical conditions [38]. The proposed method reproducibility test was performed 100 times with a 10 mg/L NO_3_-N working solution. The sampling interval was 1 s. The record values were shown in Figure 10. All data points fluctuated within the range of 9.89–9.94 mg/L. There was an error of approximately 0.1 mg/L from the standard value of 10 mg/L. This effect mainly depended on the accuracy of the mathematical model and had nothing to do with the performance of the instrument itself. The relative standard deviation (RSD) was 1.34%. The smaller the RSD value, the more concentrated the sensor’s measurement data. The results indicate that the transducer has high repeatability.

**Figure 11:**
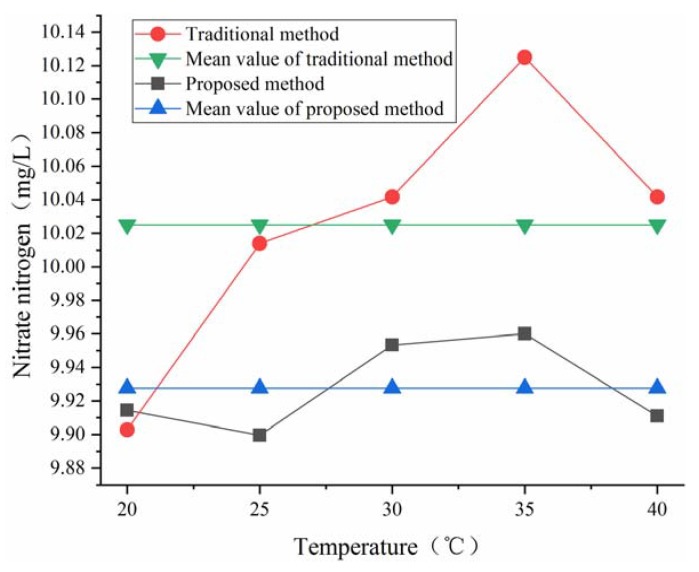
Environmental adaptability test at different temperatures.

When the transducer device is used in an outdoor environment, the temperature drift of the sensor should be considered. By analyzing the sensor’s manufacturing and measurement process, we found that the following aspects may affect the temperature characteristics of the instrument: electronic components, LEDs, photodiodes and molecular motion in a solution. The environmental adaptability of the transducer device was evaluated with several determinations of 10 mg/L NO_3_-N working solution at five different temperatures. We put the plastic cuvettes and the transducer device into the high-low temperature test chamber at the same time. The temperatures of the test chamber were set at 20 °C, 25 °C, 30 °C, 35 °C and 40 °C in turn. We kept one hour at each temperature point and finished all measurements within 6 h. To avoid the surface condensation of the photodiodes, LEDs, optical beam splitter and plastic cuvettes, it was necessary to control the relative humidity of the experimental environment in the test chamber to be below 20%. The measured data sequences were shown in Figure 11. From the test results, we observed that both of the methods suffered data fluctuations with an increase in temperature. However, the proposed method contributed to the smallest relative standard deviation (traditional method: 8.00%, new method: 2.72%). The experiment proved that the new method has the corrected function to operation conditions.

### 3.4. Field Sample Analysis

The transducer device was used on a variety of samples, both freshwater and seawater, in RAS. We added a small amount of chloroform into all samples during storage to prevent deterioration. Usually, water samples need to undergo static clarification and filtration before testing. For samples with a concentration greater than 10 mg/L, the dilution treatment is needed. The results of each sample were obtained based on five parallel experiments. The determination data were listed in Table 3. Among these water samples, NO_3_-N concentration in the shrimp tank was relatively high due to frequent feeding and strong metabolizable action. The golden trout tank water was taken from deep well water. It contained a low concentration of NO_3_-N. All measured results were in line with the relevant technical specifications for its content limit. The recovery rate of the water body ranged from 98.1–105.7%. It shows that the transducer device has high precision and is suitable for the determination of many types of water samples.

## 4. Conclusions

In summary, a novel approach to accurately detect NO_3_-N in complex aquaculture water was described, which was based on the chromogenic principle of nitrate nitrogen derivatives. In the method, the addition of ammonium sulfamate and silver sulfate eliminated the interference of coexisting nitrite and chloride ions. Using the modulated LEDs and a synchronous detector rather than a constant (DC) source, we eliminated measurement errors due to ambient light and low frequency noise and provided higher accuracy. Compared with the halogen–tungsten lamp source and the built-in desktop spectrophotometer, the modulated source had better temperature stability and longer service life. The hand-held device was powered by batteries and free from industrial electricity interference. Another advantage embodied by a hand-held device was the environmental correction function. When used for field measurement, the calibration curve does not need to be revised again. The experiments showed that the transducer device presents a higher sensitivity and a lower detection limit than the traditional single element method. The precision and sample recovery rate can meet the analysis requirements of conventional and micro scales. In addition, this low cost hardware platform will be applicable to more chemical analysis and environmental monitoring instruments. Users only need to change the primary–secondary wavelength LEDs according to the absorption characteristics of the chromatic liquid. The optical measurement module designed in this paper can also be extended to be an automatic analyzer. Our future work will focus on replacing manual sampling and dispensing processes with the automatic continuous flow injection analysis module, and a host computer will be able to remotely control the related equipment in RAS through the Internet of Things wireless transmission technology. The new integrated system can achieve the goal of reducing the nitrate level by closed loop control.

## Figures and Tables

**Table 1:** The function configuration of ADG733.

LED_CLK_LED1/2 Level	LED_CLK Level	Vin Range	Transfer Function
High	High	2.50–3.75 V	V_o_ = V_in_
Low	Low	1.25–2.50 V	V_o_ = 2 V_ref_ − V_in_

**Table 2:** Parameter description of traditional model and proposed model.

Symbols	Traditional Model (See Equation (7))	New Model (See Equation (3))
$t_{1},\cdots ,t_{n}$	$t_{n}=x_{n}$	$t_{n}=\mathrm{lg}x_{n}$
$y_{1},\cdots ,y_{n}$	$y_{n}=\log\frac{V_{R420}(n)}{K_{1}V_{S420}(n)}$	$y_{n}=\log m_{n}$
$b_{0},b_{1}$	$b_{0},b_{1}$	$b_{0}=\beta ,b_{1}=\log\alpha$

**Table 3:** Test results of sample recovery rate.

Sample Source (100 mL)	Adding Standard Matter Amount	Determination Value (mg/L)	Sample Concentration (mg/L)	Recovery Rate
*Cynoglossus semilaevis* tank	0	1.932	1.932	105.1%
	10 mL of 20 mg/L	3.667		
Tiger grouper tank	0	0.857	0.857	103.9%
	10 mL of 10 mg/L	1.724		
Shrimp tank	0	2.690	2.690	105.7%
	10 mL of 30 mg/L	5.328		
Golden trout tank	0	0.522	0.522	98.1%
	10 mL of 10 mg/L	1.366		
